# Effects of Strawberry Tree (*Arbutus unedo* L.) Aqueous Leaf Extract and Arbutin on PK-15 and HepG2 Cells

**DOI:** 10.3390/toxics12090628

**Published:** 2024-08-26

**Authors:** Vesna Benković, Ines Tkalčec, Anica Knežević, Karlo Jurica, Fabijan Knežević, Irena Brčić Karačonji, Nevenka Kopjar

**Affiliations:** 1 Faculty of Science, University of Zagreb, 10000 Zagreb, Croatiaanica.horvat.knezevic@biol.pmf.hr (A.K.); 2 Special Security Operations Directorate, Ministry of the Interior, 10000 Zagreb, Croatia; juricakarlo@gmail.com; 3 School of Medicine, Catholic University of Croatia, 10000 Zagreb, Croatia; knezevicfabijan@yahoo.com; 4 Institute for Medical Research and Occupational Health, 10000 Zagreb, Croatia; ibrcic@imi.hr (I.B.K.); nkopjar@imi.hr (N.K.); 5 Faculty of Health Studies, University of Rijeka, 51000 Rijeka, Croatia

**Keywords:** *Arbutus unedo* L., arbutin, leaf extract, MTS assay, alkaline comet assay, cytotoxicity

## Abstract

The antioxidant properties of the leaves of the Mediterranean strawberry tree (*Arbutus unedo* L.) are mainly attributed to the main bioactive compound, the phenolic glycoside arbutin. In this study, the safety profile of strawberry tree aqueous leaf extract (STE) and arbutin at the DNA level was assessed in vitro using porcine PK-15 kidney cells and HepG2 cells derived from human hepatomas. To examine the effects on cell viability and DNA damage, cells were treated for 24 h with STE or arbutin at three concentrations presumed to be non-toxic (400, 200, and 11.4 µg/mL). Assessments were performed using the MTS viability assay, dual acridine orange/ethidium bromide fluorescent staining, and alkaline comet assay. Results showed that the highest concentration (400 µg/mL) of both tested compounds had no significant cytotoxic effects on either PK-15 or HepG2 cells. Apoptosis was the predominant type of cell death and the total amount of DNA damage in treated cells was within acceptable limits. These results on the in vitro cytocompatibility of arbutin and STE with PK-15 and HepG2 cells could serve to make more reliable judgements about safe levels of arbutin in cosmetic products and functional foods, given the increased popularity of the compound in recent years.

## 1. Introduction

The beneficial effects of the strawberry tree (*Arbutus unedo* L.) have long been known. Virtually all parts of the plant (leaf, fruit, bark, root) are used in traditional medicine for the treatment of various diseases [1]. Root and bark preparations are used in folk medicine primarily for gastrointestinal disorders, cardiovascular diseases, and urological and dermatological problems [2,3]. The fruit and leaf extract of the strawberry tree is particularly useful in the treatment of urological and gastrointestinal problems due to its diuretic, uroantiseptic, and laxative effects [2,4,5]. The antioxidant effect of the fruits and leaves of the strawberry tree is stronger than that of green tea or blueberries [6,7].

Strawberry tree leaf extract has antimicrobial properties, giving rise to possible applications in the treatment of asymptomatic urinary tract infections [4]. Due to its antioxidant capacity, it can be used clinically as a preventive or therapeutic agent in diseases caused by oxidative stress [8,9,10]. Arbutin is a hydroquinone glycoside with antimicrobial properties and helps the plant to fight bacteria. It is considered the main bioactive compound in strawberry tree leaves [11]. Since both strawberry tree leaves and arbutin show antioxidant and antimicrobial properties, they can be used in food production, and the pharmaceutical and cosmetic industries [12,13].

In vitro studies have confirmed the antioxidant effect of arbutin, which goes hand in hand with its anti-cancer and anti-inflammatory effects against various toxic substances [14,15,16]. Previous research has observed high biocompatibility of strawberry tree leaf extract and arbutin with human peripheral blood lymphocytes in vitro [17], in addition to examining the biological effects of both compounds in the rat model [18,19]. The results of these studies indicated mild DNA-damaging effects in the liver and kidney cells, meriting further research. To further characterise the safety profile of both compounds at the DNA level, two cell models were selected for in vitro study: porcine PK-15 kidney cells and HepG2 cells derived from human hepatomas. The non-harmful effect previously observed in a lymphocyte model [17] led to the hypothesis that similar effects could also be expected in other cell lines, such as PK-15 and HepG2. Therefore, this study aimed to reveal new information regarding the cyto-/genotoxic effects of STE and arbutin in these cell lines, as such effects should be well documented prior to any future use and development of arbutin-containing products as dietary supplements.

## 2. Materials and Methods

### 2.1. Materials

All chemicals and reagents used in this study were purchased from Sigma-Aldrich (Steinheim, Germany) unless otherwise stated.

Strawberry tree leaves were collected on the island of Mali Lošinj, Croatia (GPS coordinates: 44°31′50″ N; 14°28′06″ E; elevation 14 m), air-dried, grinded, and extracted with water as described previously [4,17].

Phytochemical composition of STE was previously described. More detail on its characterisation was reported by Brčić Karačonji et al. [20], and here we briefly state a total of 60 phenolics including arbutin that were detected in STE, with hyperoside and flavan-3-ols as the predominant compounds. Arbutin content in the lyophilisate of STE (1.07%) was also previously determined by high-performance liquid chromatography (HPLC) with diode array detection (DAD), and the corresponding result was published by Jurica et al. [11].

### 2.2. Cells

Porcine kidney epithelial cells (PK-15) and hepatocellular carcinoma cell lines (HepG2) were obtained from ATCC (www.lgcstandards-atcc.org, accessed on 10 June 2024) and maintained at 37 °C and 95% humidity in an atmosphere with 5.0% CO_2_ (Heraeus Hera Cell 240 incubator, Langenselbold, Germany). PK-15 cells were grown in RPMI-1640 (Gibco, Invitrogen, Burlington, ON, Canada), HepG2 cells in EMEN (Sigma-Aldrich) culture medium supplemented with 10% foetal bovine serum (biosera, Cholet, France), and 1% of the antibiotic and antimycotic solution (containing 10,000 U/mL penicillin, 10 mg/mL streptomycin, 25 µg/mL amphotericin B) (Lonza Group Ltd., Basel, Switzerland). Cells were cultured to a confluence of 80–90%, which was monitored using an inverted microscope (Diavert, Leitz, Germany).

Cells were seeded at a density of 1.0 × 10^4^ cells/well on a 96-well plate (#3596, Corning^®^, Glendale, AZ, USA) and incubated for 24 h prior to treatment.

### 2.3. Treatment Protocol

To examine the effects on cell viability and DNA damage, HepG2 and PK-15 cells were treated for 24 h with strawberry tree aqueous leaf extract (STE) or arbutin at three concentrations presumed to be non-toxic (400, 200, and 11.4 µg/mL). Corresponding untreated controls were kept under the same conditions, and all treatments were performed in triplicate for each specific method. The lowest concentration of 11.4 µg/mL was calculated on the basis of the maximum allowable daily intake of arbutin (i.e., 800 mg) into the human body average weight of 70 kg. Two higher tested concentrations were determined according to previous safety assessments of arbutin-containing bearberry leave preparations as reported by the European Medicines Agency [17].

Assessments for all compounds were performed using the following methods: (1) MTS viability assay, (2) dual acridine orange/ethidium bromide fluorescent staining, and (3) alkaline comet assay. Both methods of assessing cell viability should provide reliable data on the cytotoxicity of these two compounds, while the fluorescence assay indicates the predominant type of cell death. The alkaline comet assay indicates the degree of DNA instability following treatment. 

### 2.4. MTS Assay

The MTS assay was performed according to the manufacturer’s protocol (CellTiter 96^®^ AQueous One Solution Cell Proliferation Assay (MTS); Promega, Madison, WI, USA). The absorbance values were measured using an ELISA reader (Bio-Rad Model 550 Microplate Reader, Bio-Rad, Hercules, CA, USA) at a wavelength of 490 nm. The percentage of viable cells was expressed relative to the untreated control, whose value was considered 100%.

### 2.5. Fluorescent Viability Assay with Ethidium Bromide and Acridine Orange (EtBr/AO) Staining

**Figure 1:**
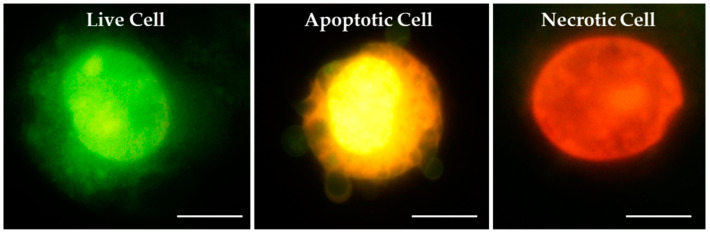
Morphological features of cells observed under epifluorescence microscope after dual ethidium bromide/acridine orange (EtBr/AO) staining. Magnification ×1000, scale bar 10 μm.

The staining procedure basically followed Harakeh et al. [21], with slight adjustments. To prepare the slides for microscopic analysis, the aliquot of a specific cell suspension (V = 20 µL) was mixed with 10 µL fluorescent dyes [100 µg/mL EtBr and 100 µg/mL AO dissolved in PBS (1:1 *v*/*v*)], and pipetted onto a slide. Preparations were immediately analysed under an epifluorescence microscope (Olympus BX51, Tokyo, Japan; 400× magnification). A total of 200 cells per sample and experiment were scored. Quantification was performed by determining the percentage of viable, apoptotic and necrotic cells. Morphological differentiation was based on differential DNA/cytoplasmic staining. Viable cells had a light green nucleus due to the incorporation of AO into their DNA. Dead cells had a dark orange/red nucleus, which was due to the binding of EtBr in their DNA. Apoptotic cells showed fragmented, green nuclei in the early phase and orange colouration of the cytoplasm in late apoptosis. Morphological features of cells after EtBr/AO staining are shown in Figure 1.

### 2.6. Alkaline Comet Assay

The alkaline comet assay procedure followed Singh et al. [22] with slight modifications as described by Jurica et al. [17]. Slides were stained with 100 µL EtBr (20 µg/mL) for 10 min. The level of DNA damage in individual cells was assessed with Comet Assay IV^TM^ software (version number: Comet Assay 4.2, TE4H-V245-UXIU-KF5N, Instem-Perceptive Instruments Ltd., Bury Saint Edmunds, UK) using an epifluorescence microscope (Olympus BX51, Tokyo, Japan) equipped with appropriate filters, under 200× magnification. A total of 200 randomly selected comets (4 × 50) were measured per each concentration (or control) tested. Tail intensity (i.e., DNA% in tail) and tail length (in micrometres) were the selected descriptors of DNA damage. The obtained results were interpreted in line with Collins et al. [23].

### 2.7. Statistical Analysis

Data were analysed using the software Statistica–Data Science Workbench, version 14.0.0.15. (TIBCO Software Inc., Palo Alto, CA, USA). Mean, standard deviation, standard error, median, minimum and maximum values were determined as basic descriptive statistical parameters. Comparisons between the values obtained for cell viability [(EtBr/AO) staining] were performed using Pearson’s χ^2^ test.

The data obtained with the alkaline comet assay were logarithmically transformed to normalise the distribution. Multiple comparisons between groups in the comet assay and MTS assay were tested using ANOVA with Tukey’s HSD post hoc test. The level of statistical significance was set at *p* < 0.05.

## 3. Results

### 3.1. Cell Viability Determined by MTS Assay

#### 3.1.1. Viability of PK-15 Cells

**Figure 2:**
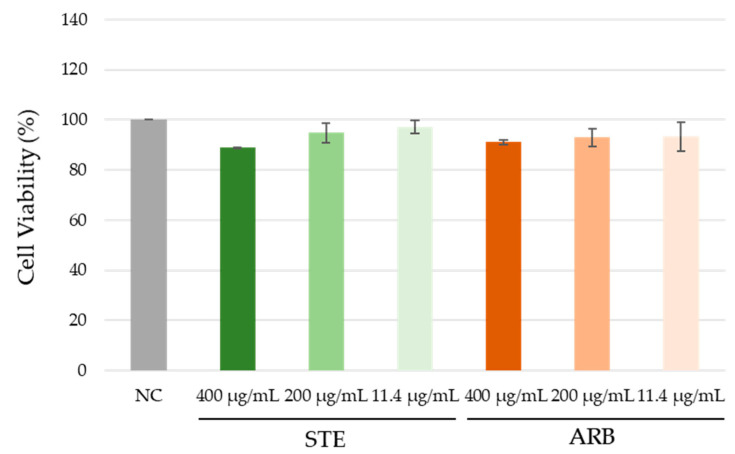
Effect of the aqueous extract of strawberry tree leaves (STE) and arbutin (ARB) on the viability of PK-15 cells after 24 h treatment in vitro. Cell samples were processed in triplicate. Viability was determined using the MTS assay, and the percentage of viability was expressed relative to the control (whose value was considered 100%). Values are expressed as mean ± standard deviation. NC—untreated control. Comparisons between the values obtained for cell viability were performed using ANOVA with Tukey’s HSD post hoc test. No difference was significant at *p* < 0.05.

The cell viability results obtained with the MTS assay are shown in Figure 2. The viability of PK-15 cells after exposure to both tested compounds did not differ significantly from the negative control (ANOVA with Tukey’s HSD post hoc test, at *p* < 0.05).

#### 3.1.2. Viability of HepG2 Cells

**Figure 3:**
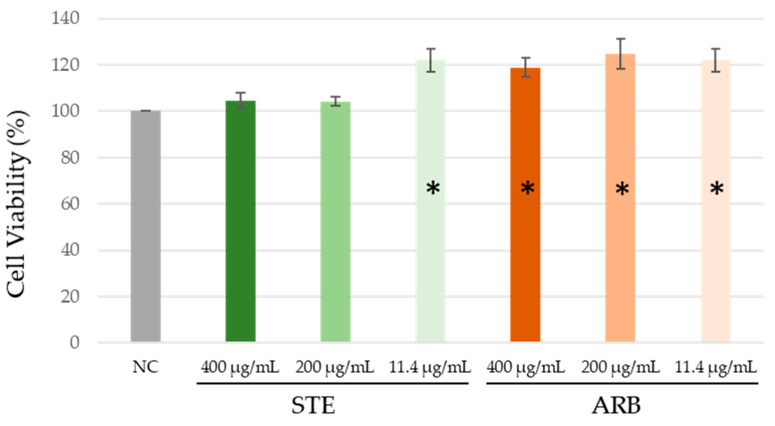
Effect of the aqueous extract of strawberry tree leaves (STE) and arbutin (ARB) on the viability of HepG2 cells after 24 h treatment in vitro. Cell samples were processed in triplicate. Viability was determined using the MTS assay, and the percentage of viability was expressed relative to the control (whose value was considered 100%). Values are expressed as mean ± standard deviation. NC—untreated control; * significantly increased compared to NC (ANOVA with Tukey’s HSD post hoc test; at *p* < 0.05).

As shown in Figure 3, none of the treatments led to a reduction in HepG2 cell viability. Interestingly, exposure to the lowest tested STE concentration and all the tested arbutin concentrations resulted in significantly higher cell viability than the negative control (ANOVA with Tukey’s HSD post hoc test; at *p* < 0.05). There are two possible reasons for this random experimental fluctuation and stimulation of cell proliferation by the treatment.

### 3.2. Fluorescent Viability Assay with Ethidium Bromide and Acridine Orange (EtBr/AO) Staining

#### 3.2.1. PK-15 Cells

**Figure 4:**
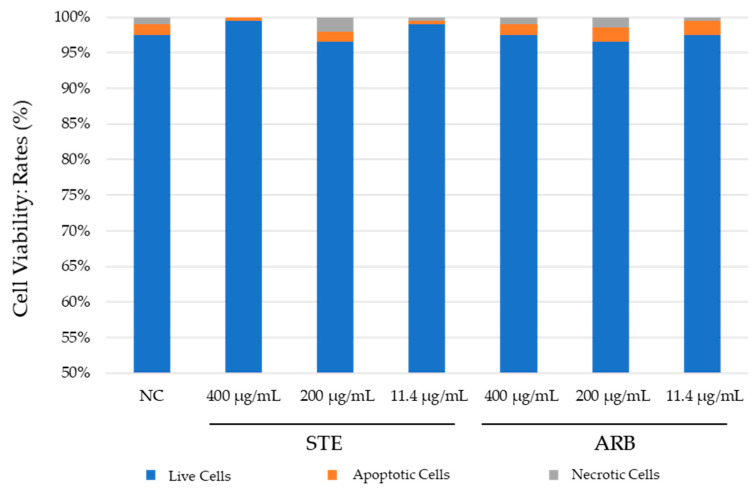
Results of fluorescent viability assay on PK-15 cells treated with aqueous extract of strawberry tree leaves (STE) and arbutin (ARB) for 24 h. NC—untreated control. Comparisons between the values obtained for cell viability were performed using Pearson’s χ^2^ test. No difference was significant at *p* < 0.05.

The results obtained with PK-15 cells using dual ethidium bromide/acridine orange (EtBr/AO) staining are shown in Figure 4. Both compounds tested showed negligible cytotoxic potential. None of the values differed significantly from the negative control (Pearson’s χ^2^ test; at *p* < 0.05). Exposure to the lowest concentration of STE (11 µg/mL) resulted in higher cell viability compared to the negative control, which could indicate a cytoprotective effect, though this requires further testing and verification by other methods (Figure 4). Apoptosis was generally the predominant type of cell death.

#### 3.2.2. HepG2 Cells

**Figure 5:**
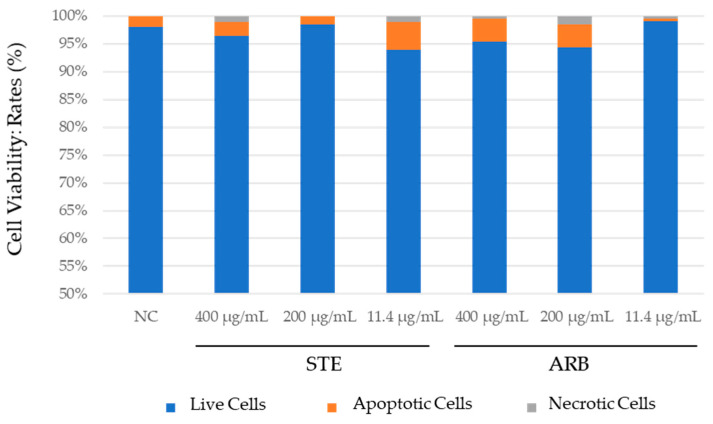
Results of the fluorescent viability assay on HepG2 cells treated with the aqueous extract of strawberry tree leaves (STE) and arbutin (ARB) for 24 h. NC–untreated control. Comparisons between the values obtained for cell viability were performed using Pearson’s χ^2^ test. No difference was significant at *p* < 0.05.

Figure 5 shows the results of the fluorescence viability assay with ethidium bromide and acridine orange for HepG2 cells. Both tested compounds showed a low cytotoxic potential. No association was found between the cytotoxic effect and the concentration of the tested substances. Exposure to the lowest concentration of STE (11 µg/mL) resulted in lower cell viability than in the negative control (Figure 5) (Pearson’s χ^2^ test; at *p* < 0.05). Most cell death was by apoptosis.

### 3.3. Alkaline Comet Assay

#### 3.3.1. PK-15 Cells

**Figure 6:**
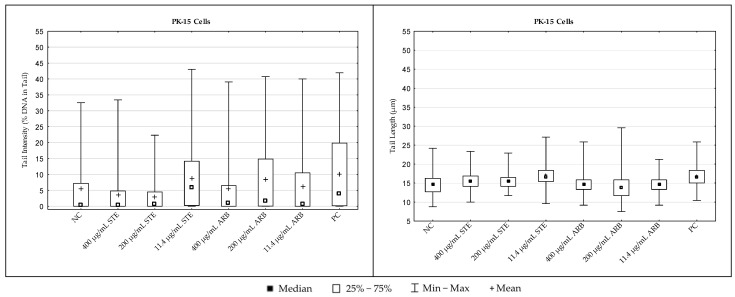
DNA damage in PK-15 cells treated with strawberry tree water leaf extract (STE) and arbutin (ARB) in vitro for 24 h and in the negative control (NC) sample, as measured by the alkaline comet assay. For each sample, two hundred independent comet measurements were performed per sample and experimental point. Tail intensity and tail length were chosen as the main descriptors for DNA damage. Hydrogen peroxide (H_2_O_2_, 100 µM) applied for 10 min served as a positive control (PC). Results are expressed as mean/median, interquartile range, and range of measured values (min–max).

The results regarding the extent of DNA damage in PK-15 cells measured with the alkaline comet assay are shown in Figure 6. Detailed inter-group comparisons and their statistical significance (ANOVA with Tukey’s HSD post hoc test, at *p* < 0.05) are reported in Table 1. For comet descriptor tail intensity, the critical F value was 12.4379, while for the other descriptor (tail length), it was 38.1667.

Considering the values of both comet descriptors, 24 h exposure to simple arbutin at all the tested concentrations did not result in significant DNA damaging effects in PK-15 cells. Exposure to STE resulted in a significant increase in mean tail intensity compared to the negative control only at the lowest concentration tested. For mean tail length, significantly increased values were observed compared to the negative control after exposure to all the tested concentrations. The positive control sample had the highest values for both comet descriptors.

#### 3.3.2. HepG2 Cells

**Figure 7:**
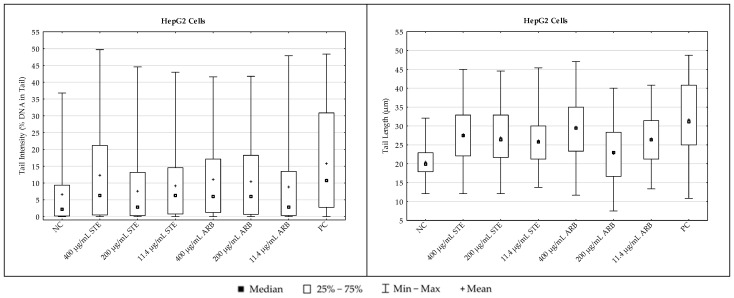
DNA damage in HepG2 cells treated with strawberry tree aqueous leaf extract (STE) and arbutin in vitro for 24 h and in the negative control (NC) sample, as measured by the alkaline comet assay. For each sample, two hundred independent comet measurements were performed per sample and experimental point. Tail intensity and tail length were chosen as the main descriptors for DNA damage. Hydrogen peroxide (H_2_O_2_, 100 µM) applied for 10 min served as a positive control (PC). Results are expressed as mean/median, interquartile range, and range of measured values (min–max).

The results regarding DNA damage levels in HepG2 cells, measured with the alkaline comet assay, are shown in Figure 7. Detailed inter-group comparisons and their statistical significance (ANOVA with Tukey’s HSD post hoc test, at *p* < 0.05) are reported in Table 2. For the comet descriptor tail intensity, the critical F value was 8.9588, while for tail length, it was 49.9409.

Looking at the tail intensity values, 24 h exposure to the highest tested concentrations of STE and arbutin resulted in a significant increase in DNA damage compared to the negative control. For mean values of tail length as another important comet descriptor, all other values were significantly higher compared to the negative control with the exception of cells exposed to 200 µg/mL arbutin (Table 2). The positive control sample had the highest values for both comet descriptors (Figure 7).

**Figure 8:**
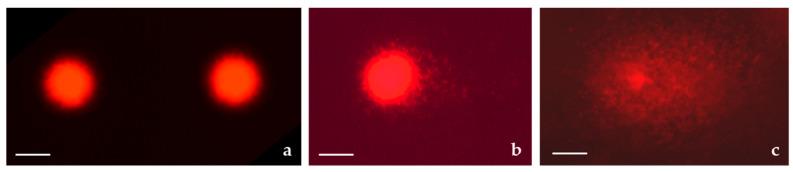
Photomicrographs of nuclei observed under an epifluorescence microscope after the alkaline comet assay. (**a**) Non-damaged DNA of HepG2 cells; (**b**) HepG2 cells treated with 400 μg/mL of strawberry tree aqueous leaf extract (STE) in vitro for 24 h; (**c**) highly fragmented nucleus of HepG2 cell in the positive control (hydrogen peroxide, 100 µM) applied for 10 min. Stained with ethidium bromide. Magnification ×200; scale bar, 20 μm.

Typical photomicrographs of the HepG2 nuclei observed after the alkaline comet assay procedure are shown in Figure 8.

## 4. Discussion

Previously observed inconsistencies in the levels of DNA damage measured in liver and kidney cells of rats exposed to arbutin or STE [18,19] indicated a need for further in-depth study of their effects. To minimise the total number of rats involved in the experiments, most studies examine exposure to only one dose of the tested compounds, and therefore, the next step was to conduct tests with a wider range of concentrations of arbutin and STE. To avoid the unnecessary use of animals in the search for effective concentrations that might be of interest, this study focused on cell models.

The results obtained confirmed the initial hypothesis and suggest that 24 h exposure to arbutin or STE in the concentration range of 11.4–400 µg/mL resulted in high cytocompatibility with PK-15 and HepG2 cells. This represents novel information, which was not known before. The significance of the results obtained with all methods is briefly discussed below.

The high cell viability observed in both PK-15 and HepG2 cells after exposure to arbutin or STE is comparable to results obtained for human peripheral blood lymphocytes [17]. Therefore, the tested substances in the concentration range 11.4–400 µg/mL (i.e., 14.9–1469 µmol/L) can be considered non-harmful in vitro, at least for three cell types of different origin: HepG2 cells of human hepatoma (tumorigenic) origin [24], PK-15 cells derived from the kidney of an adult Hampshire pig [25], and primary human blood cells derived from a healthy male donor [17].

The literature on the cytotoxic potential of STE or similar arbutin-rich multicomponent extracts is limited. In addition to previous research by this team [17], Noikotr et al. [26] tested the cytotoxicity of hexane and ethanolic leaf extracts of different members of the genus Artocarpus on human peripheral blood mononuclear (PBM) cells. They demonstrated that a 4 h exposure to the ethanolic extract of Artocarpus lacucha (containing 21% arbutin) resulted in a 50% decrease in PBM viability. Since exposure to the hexane extract of the plant did not cause such a decrease in cell viability, it appears that the nature/properties of the solvent played an important role in the observed cytotoxic effects. This was well supported by our observations as the STE tested here was prepared with water and the cytotoxicity estimated after the treatments did not differ significantly from the control values.

Far more data are available for the effects of arbutin. This compound is generally considered to be a cytoprotective rather than a cytotoxic agent [16]. Recent in vitro studies on arbutin have used different exposure scenarios and concentrations. Hazman et al. [27], using the same cell type (HepG2) and exposure time, found that 24 h exposure to 22 µmol/L α-arbutin did not result in cytotoxic effects. The concentration they tested falls within the range tested here. Sivasangari et al. [28] used the H9c2 cell line (cardiomyoblasts) and exposed them to arbutin at 10–50 μg/mL for 24 h. They found that arbutin was not cytotoxic up to a concentration of 40 μg/mL, while the viability of the cells was slightly reduced after exposure to the highest concentration These tested concentrations fall within the range tested here. The fact that viability decreased at a concentration of 50 μg/mL suggests a possible higher innate sensitivity of this type of cells compared to the HepG2 cells we used in the experiment. Finally, Ma et al. [29] observed no significant difference in the viability of adult human retinal pigment epithelial cells (ARPE-19) after 24 or 48 h of exposure to arbutin at concentrations of 25, 50, or 100 μmol/L compared to untreated controls. The tested concentrations were also comparable to those in the present study. Overall, this study showed a high degree of agreement with at least three reported in vitro approaches. However, a novelty of this study is that the highest concentration tested (i.e., 400 μg/mL, which corresponds to almost 1500 µmol/L) was not significantly cytotoxic in any of the tested cell models.

A closer look at the percentage of cells undergoing apoptosis and necrosis after all treatments revealed apoptosis was the leading type of cell death. This indicates that exposure to the two tested substances did not lead to an increased risk of necrosis-related inflammation. In general, necrosis due to its uncontrolled and chaotic nature would be a much less desirable type of cell death, especially when triggered after exposure to herbal compounds that could potentially be used in future phytotherapy applications.

The triggering of apoptosis after exposure to the tested compounds shows a highly regulated self-killing of cells that suffered irreparable DNA lesions or other types of complex cytogenetic damage. The latter type of damage was not examined in this study since the alkaline comet assay specifically detects only “primary” DNA lesions, not cytogenetic results due to disrupted DNA repair pathways or damage that has not been properly repaired. Another reason why the comet assay was selected instead of standard cytogenetic tests is that many cell lines often have some degree of chromosomal instability due to frequent in vitro divisions and adaptation to almost unlimited growth in culture, thereby limiting their use for chromosomal damage testing. HepG2 cells are known to have a hyperdiploid karyotype, containing many stable chromosomal abnormalities, and in some cases, can contain more than 100 chromosomes [24]. Similar findings have recently been reported for the PK-15 cell model [25], which has aneuploidies and complex structural variants in its genome. These shortcomings of the cell lines used in this study can also be seen as limitations of the current experimental design. However, in previous experiments using primary cells (i.e., peripheral blood lymphocytes) for testing and detailed cytogenetic analysis [17], no potentially harmful chromosomal instabilities were detected after exposure to the same concentrations of arbutin or STE.

Regarding the apoptotic potential of STE in vitro, the literature provides no other comparable data to discuss the results, with the exception of [17]. Since the fluorescent EtBr/AO assay allows morphological differentiation of apoptotic cells, the mechanisms responsible for STE-induced apoptosis cannot be defined, and given the complex composition of the extract, it can be surmised that multiple mechanisms may be involved. The tested STE was confirmed to contain sixty phenolics, with arbutin, hyperoside (quercetin-3-O-galactoside, Q3G), and flavan-3-ols as the predominant compounds [20].

When discussing the mechanisms responsible for cell death after exposure to simple arbutin, the indirect mechanisms mediated by its metabolic conversion to hydroquinone should be stated. This compound has been confirmed as an effective apoptosis inducer in several studies. Luo et al. [30] demonstrated that 24 h exposure of TK6 cells to hydroquinone at concentrations of 2.5, 5, 10, or 20 µmol/L led to apoptosis through PARP-1 via Fas upregulation (caspase-dependent pathway) and p53-mediated apoptosis. Yang et al. [31] reported apoptosis mediated by the caspase 9/3-dependent pathway in human neutrophils and eosinophils exposed to 50 μmol/L hydroquinone for 12 or 24 h. Using the HL-60 cell model, Terasaka et al. [32] investigated the apoptotic effects of 24 h exposure to hydroquinone in the concentration range of 0.0039–4 mmol/L. Their results indicated that hydroquinone activates the intrinsic apoptosis pathway via the release of cytochrome c and the activation of procaspase-3 and -9. Shen et al. [33] treated human embryonic kidney cells HEK293 with hydroquinone at 100, 200, 300, or 400 µmol/L for 24 h, and showed that the onset of apoptosis was related to the depletion of intracellular thiol. It is known that up to 70% of arbutin can be converted to hydroquinone [34,35]. However, due to extensive conjugation and rapid excretion, the amount of free hydroquinone in tissues and organs is usually less than 2% of the total administered dose [36].

The biological activities, pharmacology, pharmacokinetics, and toxicity of hyperoside and flavan-3-ols have been discussed in detail [30,37,38,39,40]. Since the hyperoside content in the leaves of the strawberry tree from which our extract was prepared was quite high (i.e., 1149.54 mg/kg dried leaf weight), this compound might also greatly contribute to the biological effects of STE in addition to arbutin, especially considering that the content of the third major constituent (rutin) was significantly lower (93.39 mg/kg dried leaf weight) [20]. Therefore, in order to discuss aspects relevant to the experimental model and to suggest possible mechanisms responsible for apoptosis, only some in vitro studies were considered, specifically reports of induction of apoptosis in different cell lines after 24 h exposure to hyperoside. These include Sudan and Rupasinghe [41], who exposed HepG2 cells to six different concentrations of Q3G (1, 10, 50, 100, 150, and 200 μmol/L) for 24, 48, or 72 h. They observed a concentration- and time-dependent inhibition of proliferation, while apoptosis was mediated by the activation of caspase-3. Sun et al. [42] investigated the effects of hyperoside on apoptosis of the human gastric cancer cell line BGC-823. After 24 h exposure to hyperoside, they determined an IC_50_ of 32.14 μg/mL, and associated apoptosis with upregulated activities of intracellular caspases-3, 8, and 9. Liu et al. [37] found that 24 h in vitro exposure of the human A549 lung adenocarcinoma cell line to hyperoside at concentrations of 20, 40, 60, 80, and 100 μg/mL resulted in concentration-dependent apoptosis via the caspase-3 and P53 signalling pathways. Using the same cell type, Yang et al. [38] observed that apoptosis after 24 h of hyperoside treatment (at 10, 50, and 100 µmol/L) was associated with activation of the p38 MAPK- and JNK-induced mitochondrial death pathway. Liu et al. [39] indicated that 24 h in vitro exposure of human thyroid squamous cell carcinoma SW579 cells to 5, 10, and 20 µg/mL induced apoptosis partly by upregulating the expression of Fas and FasL mRNAs and downregulating the expression of survivin protein. Qiu et al. [40] found concentration-dependent apoptosis in MCF-7 and 4T1 breast cancer cell lines (exposed to hyperoside at 12.5, 25, 50, 75, or 100 µmol/L for 6, 12, or 24 h) related to ROS, with a mechanism involving activation of the Bax–caspase-3 axis and inhibition of the NF-κB signalling pathway.

The present study also indicated proliferation-related problems after exposure to the tested compounds, which were more pronounced in HepG2 cells than in PK-15 cells. The lowest concentration of STE tested significantly increased the proliferation of HepG2 cells compared to the negative control. The same effect was less pronounced after exposure to the two higher concentrations. Normally, cases in which such a trend in toxicity is observed are associated with the phenomenon of hormesis [43]. Exposure to arbutin also stimulated the proliferation of HepG2 cells, but the differences between the concentrations tested and the effects observed were less pronounced than after treatment with STE. Stimulation of HepG2 cell growth was previously reported by Jurič et al. [44] after 30 min exposure to strawberry tree honey.

The extent of DNA damage measured after treatments with the alkaline version of the comet assay should also be addressed. This methodological approach can detect the broadest range of DNA lesions in individual cells [23,45,46], and can detect even subtle changes in the number of DNA breaks, from a few hundred to several thousand breaks per cell [47].

Previous reports on the genotoxicity of strawberry tree water leaf extract and arbutin in vitro suggest that both have low DNA-damaging potential [17,35]. Overall, the results from the alkaline comet assay presented here support previous reports on the cytoprotective and antioxidant properties of STE and arbutin obtained using various experimental in vitro models [17,48,49,50,51].

Although treatments with all the tested compounds resulted in measurable amounts of DNA damage in PK-15 and HpG2 cells compared to the respective controls, overall genotoxicity was relatively low. This increase in DNA damage caused by STE could be due to the complex composition of the tested extract, which is rich in polyphenolic compounds. It is well known that many phytochemicals have a dual nature with pro-oxidant and antioxidant properties [52]. Due to their pro-oxidant behaviour, they directly induce certain levels of DNA breaks and lesions that can be detected by the alkaline comet assay. Moreover, due to the imbalance between their protective and antioxidant properties, some damage is also caused by indirect mechanisms.

The DNA damage patterns determined in the present study show that the median values of tail intensity determined in PK-15 cells were generally lower than in the HepG2 model at the same concentrations tested. It is also interesting that the ranges of individual values measured for the monitored comet descriptors were more scattered in HepG2 cells than in PK-15 cells. This could be explained by the different origin of the cells, with HepG2 possibly having a slightly higher sensitivity to treatments, possibly due to metabolic differences. Greater metabolic conversion of arbutin to hydroquinone in HepG2 cells could increase toxicity and the extent of DNA damage. The results of the present study indicate that PK-15, a type of epithelial cell derived from the kidney, was slightly less damaged due to its weaker metabolising capabilities.

The results obtained showed no clear concentration-dependent effects at the DNA level. This could be related to both the sensitivity of the comet assay method and the duration of treatment. This assay effectively recognises “simple” DNA lesions such as single-strand breaks, which are also most quickly repaired. As the exposure lasted 24 h, the extent of damage actually reflected the balance that occurred between the induction of damage and its repair.

New data on the in vitro cytocompatibility of arbutin and STE with PK-15 and HepG2 cells reported here could serve as further important evidence for understanding the safety profiles of each of these substances.

## 5. Conclusions

This study established that (1) 24 h exposure to the highest concentration of both substances tested (400 μg/mL) had no significant cytotoxic effects on either PK-15 or HepG2 cells; (2) apoptosis was the predominant type of cell death; and (3) the total amount of DNA damage in treated cells was within the limits considered acceptable by the basic rules of the comet assay. A special added value of the present study is that arbutin and STE in the concentration range 11.4–400 µg/mL (i.e., 14.9–1469 µmol/L) can be considered non-harmful in vitro for HepG2 cells of human hepatoma (tumorigenic) origin and for PK-15 cells derived from the kidney of an adult Hampshire pig.

The findings of the present study provide novel information that is potentially useful in clarifying the safety profile of STE, which must be well described before any use of this plant (or its parts) in the form of a specific herbal medicine, adjuvant, or nutraceutical product. The study also contributes to the knowledge on safe levels of arbutin in food, cosmetic products, or functional foods.

Future research could be continued using suitable in vitro and in vivo experimental models. Considering that this study focused on the individual effects of arbutin and STE on two cell models, with evidence of their cytocompatibility, future studies could be raised to a higher level in which the tested concentration ranges can be combined with known cyto/genotoxic agents to establish the level of protective effect that these specific concentrations offer.

## Figures and Tables

**Table 1:** The results for statistical analysis of data measured by the alkaline comet assay in PK-15 cells treated with strawberry tree water leaf extract (STE) or arbutin (ARB) in vitro for 24 h and in the negative control (NC) sample. Significance was tested using ANOVA with post hoc Tukey’s HSD test at the level of *p* < 0.05. n. s.—not significant. PC—positive control.

Experimental Groups		Tail Intensity							
		NC	400 µg/mL STE	200 µg/mL STE	11.4 µg/mL STE	400 µg/mL ARB	200 µg/mL ARB	11.4 µg/mL ARB	PC
Tail Length	NC		n. s.	n. s.	<0.0001	n. s.	n. s.	n. s.	=0.0001
	400 µg/mL STE	=0.0035		n. s.	<0.0001	n. s.	=0.0013	n. s.	=0.0001
	200 µg/mL STE	=0.0313	n. s.		<0.0001	n. s.	=0.0006	n. s.	=0.0001
	11.4 µg/mL STE	<0.0001	<0.0001	<0.0001		=0.0005	n. s.	=0.0021	n. s.
	400 µg/mL ARB	n. s.	=0.0107	n. s.	<0.0001		n. s.	n. s.	=0.0011
	200 µg/mL ARB	=0.0168	<0.0001	<0.0001	<0.0001	=0.0058		n. s.	n. s.
	11.4 µg/mL ARB	n. s.	=0.0016	=0.0160	<0.0001	n. s.	=0.0336		=0.0048
	PC	<0.0001	=0.0003	<0.0001	n. s.	<0.0001	=0.0001	=0.0001	

**Table 2:** The results for statistical analysis of data measured by the alkaline comet assay in HepG2 cells treated with strawberry tree water leaf extract (STE) and arbutin (ARB) in vitro for 24 h and in the negative control (NC) sample. Significance was tested using ANOVA with post hoc Tukey’s HSD test at the level of *p* < 0.05. n. s.—not significant. PC—positive control.

Experimental Groups		Tail Intensity							
		NC	400 µg/mL STE	200 µg/mL STE	11.4 µg/mL STE	400 µg/mL ARB	200 µg/mL ARB	11.4 µg/mL ARB	PC
Tail Length	NC		=0.0073	n. s.	n. s.	=0.0110	=0.0253	n. s.	<0.0001
	400 µg/mL STE	<0.0001		n. s.	n. s.	n. s.	n. s.	n. s.	=0.0328
	200 µg/mL STE	<0.0001	n. s.		n. s.	n. s.	n. s.	n. s.	<0.0001
	11.4 µg/mL STE	<0.0001	n. s.	n. s.		n. s.	n. s.	n. s.	=0.0006
	400 µg/mL ARB	<0.0001	n. s.	=0.0226	=0.0002		n. s.	n. s.	=0.0239
	200 µg/mL ARB	n. s.	<0.0001	<0.0001	<0.0001	<0.0001		n. s.	=0.0101
	11.4 µg/mL ARB	<0.0001	n. s.	n. s.	n. s.	=0.0023	<0.0001		<0.0001
	PC	<0.0001	<0.0001	<0.0001	<0.0001	n. s.	<0.0001	<0.0001	

## Data Availability

All data are available in the manuscript.
